# Sex differences in SARS-CoV-2 infection rates and the potential link to prostate cancer

**DOI:** 10.1038/s42003-020-1088-9

**Published:** 2020-07-08

**Authors:** Dimple Chakravarty, Sujit S. Nair, Nada Hammouda, Parita Ratnani, Yasmine Gharib, Vinayak Wagaskar, Nihal Mohamed, Dara Lundon, Zachary Dovey, Natasha Kyprianou, Ashutosh K. Tewari

**Affiliations:** 1 Department of Urology and The Tisch Cancer Institute, Icahn School of Medicine at Mount Sinai, New York, NY 10029 USA; 2 Department of Emergency Medicine, Icahn School of Medicine at Mount Sinai, New York, NY 10029 USA; 3 Department of Oncological Sciences, Icahn School of Medicine at Mount Sinai, New York, NY 10029 USA

**Keywords:** Prostate cancer, SARS-CoV-2, Respiratory tract diseases

## Abstract

The recent outbreak of infections and the pandemic caused by SARS-CoV-2 represent one of the most severe threats to human health in more than a century. Emerging data from the United States and elsewhere suggest that the disease is more severe in men. Knowledge gained, and lessons learned, from studies of the biological interactions and molecular links that may explain the reasons for the greater severity of disease in men, and specifically in the age group at risk for prostate cancer, will lead to better management of COVID-19 in prostate cancer patients. Such information will be indispensable in the current and post-pandemic scenarios.

## Abstract (web summary)

Dimple Chakravarty et al. review the rapidly emerging data indicating a higher rate of SARS-CoV-2 infection in men. They note that men in the age group most at risk of infection are also at high risk of prostate cancer, and explore the potential links between these diseases and implications for COVID-19 treatment in prostate cancer patients.

## Introduction

The COVID-19 disease pandemic caused by the rapid spread of acute respiratory syndrome coronavirus 2 (SARS-CoV-2) infection has affected more than 7,500,000 cases and claimed more than 430,000 lives (https://coronavirus.jhu.edu). The disease has resulted in substantial individual and societal costs and presents a significant challenge to the global scientific and medical community. COVID-19 affects both sexes, and every age group and ethnicity, albeit to varying degrees. COVID-19 disease burden is disproportionately higher in men, and adverse outcomes are further compounded by older age and comorbidities.

**Fig. 1:**
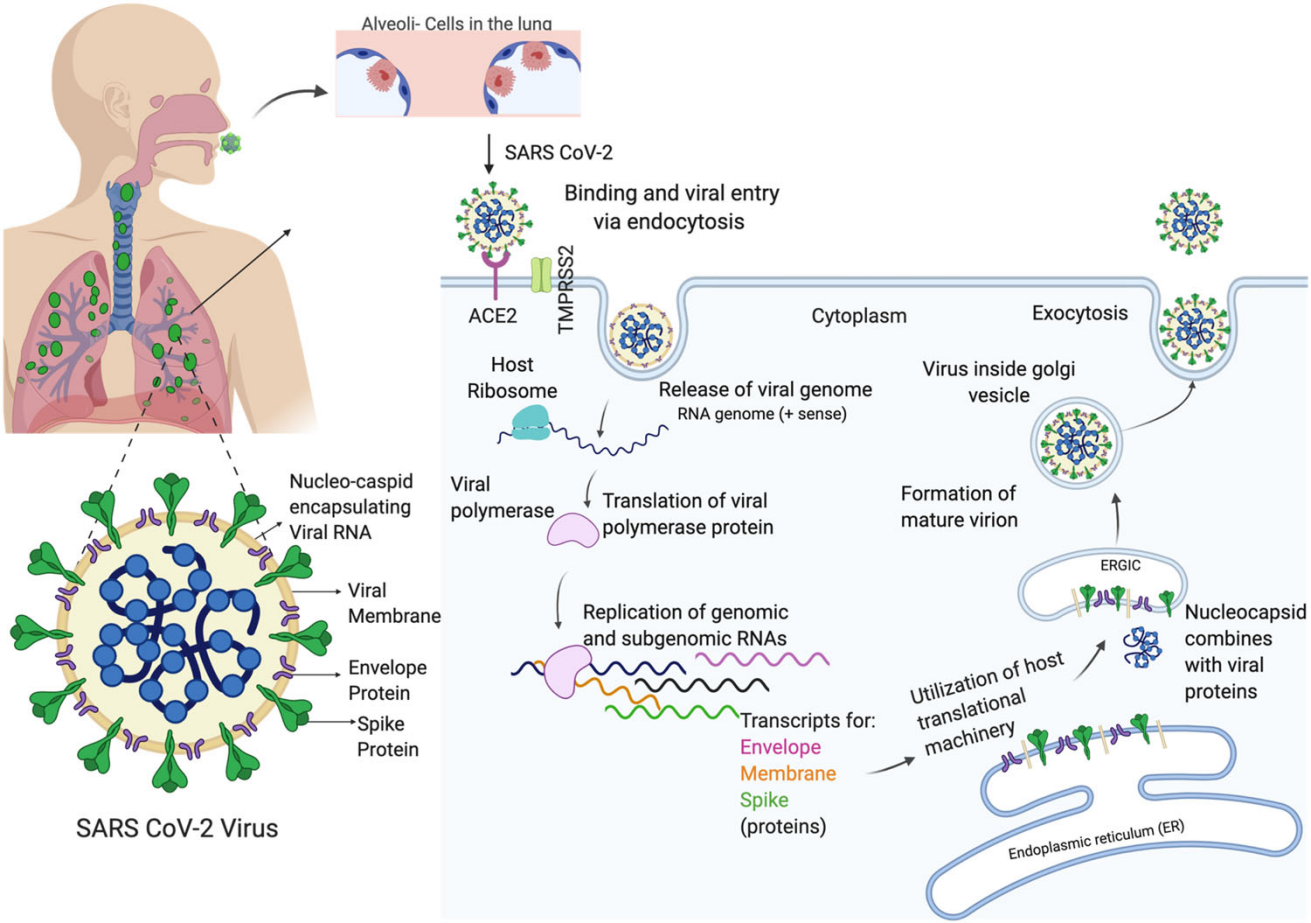
Molecular drivers of SARS-CoV-2 productive infection. ACE2 and TMPRSS2 are co-expressed on ciliated bronchial epithelial cells and type II pneumocytes and the epithelia of small intestine thus making these potential organ sites and routes for SARS-CoV-2 infection. The virus utilizes the Spike glycoprotein (S) to attach to the host cell and trigger fusion events between the virus and host lipid bilayers. Host ACE2 and TMPRSS2 are critical mediators of this process. The virus is then internalized through receptor-mediated endocytosis, or via clathrin-mediated pathways or through lipid rafts at the plasma membrane. Once inside the host cytoplasm, the viruses release their genomes to allow replication of their genetic material. Encapsulated viral-like particles with RNA genomes arise in the ERGIC complex before they erupt as mature virus.

Prostate cancer patients belong to the age group that is more susceptible to SARS-CoV-2 infection and, given their cancer, are at a higher risk of developing severe illness due to a weak immune system. Furthermore, approximately 18% of prostate cancer patients have multiple comorbid conditions^1^ that can exacerbate the risks associated with COVID-19 infection. While much is now known about how the virus gains entry into the host cells (Fig. 1) and the general disease trajectory (see Box 1), how COVID-19 infection may impact different patient populations is not yet well known. Clinicians caring for prostate cancer patients will be expected to mitigate the risks associated with COVID-19 infections while also providing the best clinical care for patients who are dealing with decisions about biopsy, active surveillance, surgery, radiation, hormonal therapy, and chemotherapy. This review summarizes recent developments and research and provides just-in-time considerations of our current understanding in three areas: the epidemiological and biological evidence for gender and/or sex disparity in COVID-19 disease; the potential association between COVID-19 and prostate cancer molecular pathogenesis; and current therapeutic options for COVID-19 patients and, in particular, COVID-19 patients with prostate cancer.

Box 1 What is SARS-CoV-2? **Structure of the virus**

Based on sequencing analyses, the novel zoonotic coronavirus SARS-CoV-2 or 2019-nCov belongs to the subfamily coronavirinae and genus, betacoronavirus. The 30 kb genome of 2019-nCOV is a single-stranded positive strand RNA (+ssRNA). Two-thirds of the genome serves as a template for non-structural proteins by direct translation into polyprotein 1a/1ab (pp1a/pp1ab). Sixteen non-structural proteins (nsps; nsp1–16) derived from pp1a/pp1ab play specific roles in the replication of CoVs and in the formation of replication–transcription complex (RTC) that facilitates synthesis of subgenomic RNAs (sgRNAs)^155,156^.

All structural proteins including the four major proteins (i.e., membrane (M), spike (S), envelope (E), nucleocapsid (N)), and all accessory proteins are derived from sgRNA^156,157^. Similar to all other CoVs, the four structural proteins are critical for virion assembly and infection of 2019-nCoV^157^. Sequencing analysis of non-structural proteins (nsps) and structural proteins indicates that non-structural proteins (nsps) are conserved among CoVs (58% identical) while a greater diversity has been observed between structural proteins of CoVs (48% identical), suggesting that the structural proteins might be driving the infectivity, adaptation, and transmission to new hosts^158^.

**Role of host cellular proteins ACE2 and TMPRSS2 in SARS-CoV-2 infection**

The structural spike (S) protein drives entry of coronaviruses SARS-CoV and SARS-CoV-2 into target host cells by engaging the cellular receptor, angiotensin-converting enzyme-2 (ACE2)^83^, an interferon-regulated gene. The interaction of SARS-CoV-2 S protein with the host ACE2 facilitates attachment of the virus to target cells^83,84^. This step is accompanied by the activation of cellular TMPRSS2, a type-II transmembrane serine protease that facilitates entry of virus into the target cell^84^. Earlier studies have shown that successful SARS-CoV infection is dependent on the proteolytic activity of TMPRSS2 and results in cleavage of the SARS S protein at multiple sites^84,159^. Proteolytic cleavage of the SARS S protein by TMPRSS2, a process called S priming, mediates efficient virus–host cell fusion and decreases virus sensitivity to neutralizing antibodies^83,84^. ACE2 and TMPRSS2 are co-expressed on ciliated bronchial epithelial cells and type II pneumocytes and the epithelia of the small intestine, thus making these potential sites and routes for SARS-CoV and SARS-CoV-2 infection. Efficient entry into the host cells requires three main steps and is summarized in Fig. 1. First, the virus binds to the host cell surface through a host receptor; secondly, the viral lipid membranes/envelope fuses with the host cellular membrane; and finally, the genome is released inside the host cell. The virus utilizes the Spike glycoprotein (S) to attach to the host cell and trigger fusion events between the virus and host lipid bilayers^83,84^. The S protein is often cleaved by host proteases into two distinct fragments that mediate binding to the receptor and membrane fusion^83,84^. Once inside the host cytoplasm, the viruses release their genomes to allow replication of their genetic material. SARS-CoV symptoms can be divided into three phases, where the initial phase of viral replication presents clinical symptoms of fever and cough, the second phase of virus progression is clinically associated with high fever and pneumonia-like symptoms, and the final phase progresses to acute respiratory distress syndrome (ARDS) and death^160^.

### Epidemiological evidence for, and biological mediators of, sex disparity in COVID-19 disease

Sex-based differences in incidence and mortality from COVID-19: The SARS-CoV-2 virus, which is responsible for COVID-19, binds to ACE receptors in the lungs, causing alveolar damage and is the central phase of disease progression^2^. Emerging global data suggest that men appear at higher risk of infection and mortality from COVID-19 with SARS-CoV-2 than women^3–13^. This sex-based disparity is seen in incidence and hospitalization, as well as in mortality from COVID-19^9^. This disparity was first reported from China, where the death rate among men was 2.8% vs. 1.7% in women^10^. Subsequently, this sex-based disparity was reported from France, Germany, Iran, Italy, South Korea, UK, and USA. A recent meta-analysis, that included 59,254 patients from 61 studies, reported higher incidence in, as well as fatality from, COVID-19 in males compared to females^11^. Italian epidemiological data suggest a 3:1 male:female ratio for SARS CoV-2 infection^9^. Data from a case series of COVID-19 patients in China indicate that the risk of death is 2.4 times higher in men than in women^12^. As of June 14th 2020, mortality by sex in New York State was reported to be 42% for females vs. 58% for males (https://www.syracuse.com/coronavirus-ny/). In a case series of COVID-19 patients hospitalized in New York City, 60.3% of inpatients were male, 66.5% of inpatients who required ICU admission were male, and mortality rates were consistently higher for males across all age groups older than 20 years^13^. Emerging data from multiple countries indicate higher incidence and mortality of COVID-19 in men (Table 1).

**Table 1:** COVID-19 Incidence and fatality rates across different countries^6,152^.

Countries	Incidence		Mortality	
	Male	Female	Male	Female
USA	51	49	57	43
Spain	44	56	58	42
Russia	44	56	55	45
UK	51	49	55	45
Italy	47	53	62	38
China	51	49	64	36
India	76	24	64	36
Germany	48	52	56	44

Comorbidities contribute to sex differences in COVID-19: The relationship between COVID-19, comorbidities (e.g. hypertension, cardiovascular disease, diabetes, and obesity), behavioral factors (e.g., smoking and alcohol consumption), and sex is complex. Comorbidity accounts for 71% of hospital admissions and up to 90% of ICU admissions^14,15^. Diabetes and cardiovascular disease alone account for two-thirds of COVID-19 related ICU admissions^15^ and hypertension is seen in 50–70% of all COVID-19 hospitalizations in patients over the age of 50^14^. Along with age, cardiovascular illness predicts disease severity and ICU admissions, as do hypertension and obesity^16–22^. ICU admissions were twice as high in diabetic or hypertensive patients, and three times as high in patients with cardiovascular disease^23^. Comparison of plasma levels of ACE2 from two separate cohorts comprising of 537 women and 1485 men with heart failure, revealed that the circulating levels of ACE2 in the plasma of men were higher than levels tested in women. Based on this evidence, the study suggests that the difference in plasma ACE2 may explain the severity of COVID-19 in men^24^. A study conducted in France confirmed that obesity was a risk factor for invasive mechanical ventilation, the risk being highest in those with a BMI > 35 kg/m^2^^18^. Males have higher comorbidities than females globally. Patients with comorbidities have a higher risk of infection with SARS-CoV-2, the most common being COPD, diabetes, hypertension, and cancer (Table 2). Further, among COVID-19 positive patients the incidence of comorbidities was found to be higher in males compared to females (Table 2). Initial studies in China reported COPD, diabetes, hypertension, and cancer as a significant risk factors for worse outcomes in hospitalized patients^22^. In a study of 168 severely ill COVID-19 patients, male patients were more likely to have comorbidities and men with comorbidities were more critically ill compared to men without comorbidities^25^. In another study of 5700 COVID-19 patients in the New York City area, the predominant comorbidities in hospitalized patients were hypertension, obesity, and diabetes^13^.

**Table 2:** Incidence of comorbidity in COVID-19 patients by sex^6,40,153^.

Comorbidity	Male (%)	Female (%)
COPD	67.9	32.1
Diabetes	61.1	38.9
Hypertension	68.4	31.6
Cancer	54.72	45.28
Smoking	36.1	7

Another reason for more severe COVID-19 infection in men may be because they are more likely than women to be active smokers. Active smoking has been shown to raise ACE2 receptor expression in the lungs, which increases SARS-CoV-2 attachment and entry into alveolar epithelial cells^26,27^. We can also infer that because smoking is more common in men and because smoking leads to such comorbidities as pulmonary hypertension and chronic lung disease, being a male smoker with comorbidities may represent high risk for susceptibility to COVID-19. We note, here, however, that lung cancer is the second most common cancer in both men and women and the leading cause of cancer deaths. Further study on the effects of active tobacco use on COVID-19 will be needed to determine specific associations with other comorbidities.

Smoking alters the physiological ratio of androgen to estrogen, which can lead to the priming of TMPRSS2^28^. A retrospective cohort study of 87 Chinese patients analyzed risk factors for worse COVID-19 disease progression, including clinical characteristics, age, and history of smoking. Smokers were 14 times more likely to show worse outcomes^29^. Multiple observational studies have been conducted to affirm the association between active smoking and adverse outcomes in COVID-19, including severe symptom progression, ICU admission, and death. While no differences in mortality between smokers and non-smokers were found, the majority of the studies found that significantly higher proportions of patients with severe COVID-19 symptoms and those requiring ICU admission were active or past smokers^26,30–33^. In a systematic review, Vardavas et al. concluded that smokers were 1.4 times more likely to exhibit severe COVID-19 symptoms and 2.4 times more likely to need intensive care^27^.

Age as a factor for higher risk: The most substantial factor for susceptibility to, complications of, and mortality from COVID-19 is age^9,12,19,20,25,29,31,34^. Older age is a strong predictor of death in almost all of the published studies, regardless of the risk group under investigation (cancer patients, patients with comorbidities, ICU admissions, etc.)^9,19,20,31^. Older males had higher comorbidities than females and also higher risk of worse outcomes from the infection^12,25^. Older patients are eight times more likely to have worse disease progression^29^. In studies from China, case fatality ratios were 20 times higher in those older than 60 than those younger^34^; and twice as high for those older than 80 than those between 60–80 years of age^34,35^. The same age trend was seen when comparing the likelihood of hospitalization from COVID-19. These results are consistent with US census data^36,37^, including cumulative hospitalization rates at 89.3 per 100,000, with the highest rates in people 65 years and above^38^. The CDC lists age above 65 as the top risk factor for developing severe COVID-19 illness, followed by chronic lung disease, heart disease, immune suppression, obesity, diabetes, and kidney disease^39^.

The study of gender-based and sex-based differences in COVID-19 is a priority, since better understanding of these disparities will help in the development of better therapeutic strategies and vaccines, as well as public health policies.

Cancer and COVID-19: In addition to the other comorbidities, growing evidence suggests worse clinical outcomes for cancer patients with COVID-19. Cancer patients suffering from COVID-19 are at a higher risk for fatality from COVID-19 mainly due to their immunosuppressive state and co-existing medical conditions^40^. Initial studies from China and Italy first suggested that cancer patients are susceptible to severe forms of the disease including mortality^41–44^. Dai et al. reported in their study on patients hospitalized in Hubei Province in China, that cancer patients had a significantly higher mortality compared to patients without cancer. Further, patients with metastatic cancer were at even higher risk. Mechanical ventilations (OR 2.71, *P* < 0.04), ICU admissions (OR 3.13, *P* < 0.01), and death rates (OR 2,17, *P* < 0.06) were reported to be higher in cancer COVID-19 patients than age-matched cancer-free counterparts^40^. Another meta-analysis on a cohort from China on 44,672 patients also confirms that cancer patients were at significantly higher risk of death from COVID-19^45^. A population-wide study on 4532 Italian patients found 118 patients had prostate cancer (2.6%), and 430 (9.4%) of all patients had cancer. The study highlighted the role of sex specifically in patients with cancer; male cancer patients were 79% more likely to test positive for SARS CoV-2 (OR 1.79, 95% CI 1.62–1.98, *P* < 0.0001)^46^.

Studies on COVID-19 patients from a New York Health System reported that compared to non-cancer patients, the fatality rates due to COVID-19 were two to three times higher in cancer patients. The study also investigated the mortality rates of COVID-19 among the most common cancer types observed in the US population and found the rates to be 55% for lung cancer, 14% for breast cancer, 20% for prostate cancer, and 38% for colorectal cancer^47^. A study from the Mount Sinai Health System reported that of 5688 COVID-19 patients 6% had breast, prostate, lung, urothelial, and colon cancer with 89% higher risk of intubation than non-cancer patients^48^. The incidence of, and mortality from, COVID-19 in cancer patients, is shown in Table 3. In addition, our unpublished data suggests similar patterns of greater disease burden in men. Mount Sinai Health System data from March 1, 2020 to April 26, 2020 includes 9648 COVID-19 positive patients; 5238 (54.3%) were male, of which 114 (2.2%) had been diagnosed with prostate cancer. The rate of intubation was greater in those with a diagnosis of prostate cancer than male patients with a malignancy other than prostate cancer (25.4% and 21.3% respectively, *P* = 0.02). The mortality rate in this cohort of prostate cancer patients was also statistically greater than the rate of mortality among all male patients in the cohort with a malignancy other than prostate cancer (23.7% (*N* = 27) vs. 12.7% (*N* = 43) respectively, *P* < 0.01).

**Table 3:** Incidence and mortality rates of cancer cases with COVID-19^40,48,154^.

Cancer	Incidence (%)	Mortality (%)
Lung cancer	20.95	55
Breast cancer	10.48	14
Hematological (leukemia, lymphoma, and myeloma)	8.57	37
Prostate cancer	16.76	20
Urothelial cancer	5.38	38
Colorectal cancer	4.79	38

In summary, cancer patients are more susceptible to SARS-CoV-2 infection and are associated with more severe outcomes.

Hormonal regulation and immunological basis of sex-differences in infectious diseases: Immunological differences between males and females contribute to divergence between males and females in response to SARS-CoV-2. Men are more susceptible to virus infection and produce lower levels of antibodies than women. Women have a stronger innate immune system, which confers quick and broad protection to viral infections. Innate immune cells like macrophages, monocytes, mast cells, and dendritic cells play a vital role in this process^49–51^. Innate immune recognition involves the protein sensors of RNA viruses, such as SARS-CoV-2, which are encoded by genes belonging to the family of Toll-like receptors (TLRs) located on the X-chromosome. TLR7 and TLR8 are capable of detecting single-stranded RNA. Because of its bi-allelic expression, women have higher levels of TLR7, which contributes to stronger innate immune responses and faster clearance of the virus. In addition to TLR7, several other immune regulatory genes located on the X-chromosome (e.g., TLR8, FOXP3, CXCR3, and CD40L) contribute to stronger immune response against viruses in women^7,51–53^. Women also show higher levels of type-1 interferon genes that are critical to jump-starting innate immune response following a bacterial or virus challenge^53,54^. A sex difference is also reported with regard to viral shedding^55,56^. Time to clearance of SARS-CoV-2 was found to be significantly earlier in females compared to male COVID-19 patients^55,56^. Also within families with more than one infected family member, females cleared the virus faster^56^. A recent meta-analysis of COVID-19 patients explained key immunological differences in males and females that affect susceptibility. The study found higher prevalence of immune mediators that are associated with adverse outcomes of SARS-CoV-2 in men, including TNFSF13B, CCL14, CCL23, IL-7, IL-16, and IL-18^57^.

Differences in the activity of male and female steroid hormones could potentially impact COVID-19 disease pathogenesis, prophylaxis, and response to antiviral drugs and vaccines. Studies have shown that the steroid hormones testosterone, estrogen, and progesterone, and their respective nuclear hormone receptors, regulate downstream signaling that evokes different effects and responses from the immune system^54,58^. The prevailing model for classification of hormonal influence on immune responses suggests that testosterone and progesterone decrease immune responses while estrogens can enhance immune responses^58^. Estrogen receptors (ERs) play an essential role in the development of female reproductive physiology, and aberrant estrogen/estrogen receptor signaling is a significant oncogenic driver for breast cancer. Similar to androgens in males, estrogens, and estrogen receptors in females can alter the activity of immune cell types associated with innate and adaptive immune response^59–63^ and have been shown to be immunoprotective. Sex-specific differences in innate and adaptive responses contribute to the disparity in disease outcomes following a viral challenge. Immune cells associated with innate response, including monocytes, dendritic cells, and macrophages, are functionally more active in females^54^. Estrogens suppress the production of IL-12 from stimulated macrophages and levels of pro-inflammatory IL-6 by directly altering CD16^59,63^. In addition, estrogens also influence the levels of natural killer (NK) cells^59,60^. NK cells were found to be higher in women above age 70 when compared to men of the same age group. Women in this age group had a higher CD56bright/CD56dim ratio^59,62^. Notably, compared to males the CD56dim (mature NK) cells tested in females had more functional cytolytic activity and produced higher levels of MIP-1^54,59,62^.

A comparison of adaptive immune responses between males and females demonstrates that, females have higher levels of CD4/CD8 ratios^59^. Interestingly several proinflammatory and antiviral genes expressed by cytotoxic T cells carry estrogen receptor elements in their promoter and contribute to stronger cytotoxic response in females^54,64^.

Preclinical animal studies of SARS and MERS virus have demonstrated sex-specific differences in infectivity, with males showing a higher prevalence and severity of the infection. When challenged with the SARS-CoV virus, male mice showed elevated expression of pro-inflammatory cytokines CCL2 and IL-6 in the lungs and consequently severe pathogenesis associated with the infection^65^. In the absence of estrogens, the effects of pro-inflammatory TNF and CCL2 expression from pathogenic IMMs and neutrophils contribute to poor outcome following SARS infection in males^65^.

In the context of both SARS and MERS, males seem to be more severely affected than females^4,66,67^. Several SNPs in the epidermal growth factor (EGF) gene were associated with ARDS risk in males but no association could be predicted in females. EGF is one of the critical components in the proliferative phase of ARDS. In one study, SNPs resulted in an upregulation of EGF that may worsen ARDS in males^68^. Another study reported that males were more prone to endothelial dysfunction and that women were protected in this regard^69^. Endothelial dysfunction is key in triggering ARDS and these associations may suggest a potential mechanism of protection in females against SARS or MERS.

Distinguishing early immune activation events from later phases of the disease where the immune system is dysregulated due to chronic inflammatory signals would be key in enhancing our understanding of the disease. Study designs could include the criteria of disease stages and analyze men or women within a specific disease stage. It is also critical to consider that the outcomes of treatments may differ by sex, and sex bias would be an important parameter to consider in testing therapies in the future.

In summary, females exhibit robust innate and adaptive immune responses to viral infections. Elevated transcriptional activation of immune response genes on the X-chromosome and sex-specific steroids like estrogens, help to facilitate faster clearance of viral loads in females^70^.

### Potential association between COVID-19 and prostate cancer molecular pathogenesis

**Fig. 2:**
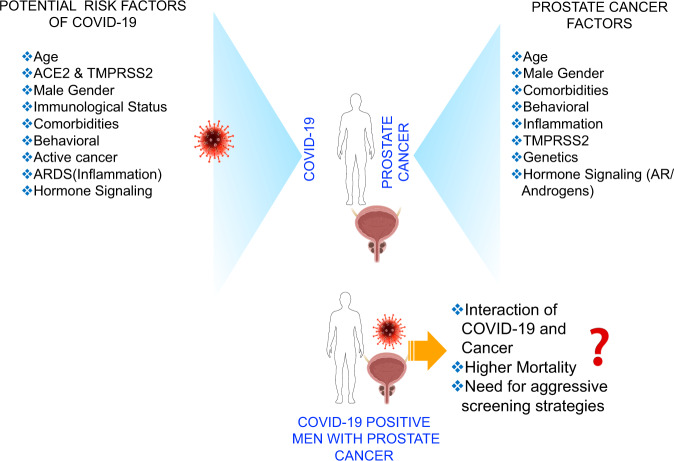
Comparison of Risk factors between COVID-19 and prostate cancer. Men are at a higher risk of SARS-CoV-2 infection than women. Shared risk factors involved in COVID-19 mortality, prostate cancer risk, and conceptual rationale for aggressive clinical management of COVID-19 in prostate cancer patients in the post-pandemic era or if the infection reoccurs.

Common risk factors for the two diseases: Accumulating evidence suggests similarities between risk factors for COVID-19 and prostate cancer (Fig. 2). Comorbidities such as hypertension, diabetes, and alcoholism, and behavioral factors like active smoking that substantially influence the severity of COVID-19^71^ are also known to influence prostate cancer progression and outcomes^1,72–75^. Interestingly, the most potent common risk factor in the two diseases is age. The risk for prostate cancer increases in men above the age of 50, and notably, this is the age group that is most susceptible to complications of, and mortality from, COVID-19^9,12,19,20,25,29,31,34,76^. Based on existing scientific knowledge, we can make a number of assumptions. First, older age and comorbidities (hypertension, diabetes, obesity, and smoking) that adversely affect COVID-19 are also lethal for prostate cancer. Second, prostate cancer patients are much more vulnerable to COVID-19 complications and risk of death due to their compromised immune status. Third, older males with prostate cancer who are active smokers, hypertensive, diabetic, and overweight are by far the most vulnerable group for contracting and dying from COVID-19.

Androgens impact immune response following viral infection: The androgen receptor (AR) gene located on the X-chromosome regulates developmental programs associated with male phenotypes^77^. Additionally, AR plays a critical role in regulating cellular programs associated with prostate physiology and homeostasis^78^. Dysregulation of androgen and AR signaling is directly associated with prostate cancer. Nuclear localization and transcriptional activation of AR is coordinated by binding to the ligands testosterone (T) or 5alpha-dihydrotestosterone (DHT)^78^. Recent studies indicate that ACE2 may be regulated by AR signaling^57,79^. Several studies have shown that androgens can impact the breadth of immune response by altering the activity of specific immune subsets that are directly involved in clearing viruses, for example, all cells derived from myeloid lineage express AR^80^. These include neutrophils, monocytes, macrophages, mast cells, and eosinophils. The immune suppressive effects of androgens on cells of the myeloid lineage are well characterized. In addition to myeloid cells, androgens also influence the functional activity of dendritic cells. In vitro studies demonstrate that androgens can suppress the expression of major histocompatibility complex (MHC)/human leukocyte antigen (HLA) and other costimulatory molecules on dendritic cells and thereby impact cytokine production and T-cell infiltration to the target area^81^. Androgens suppress B lymphopoiesis and are associated with low antibody production during vaccination^61^. Lower levels of antibody titer in males compared to females in response to infection or vaccination may be due to the underlying inhibitory effect of androgens on B cells and monocytes^58,80^. A study conducted in China reported that the concentration of the SARS-CoV-2 IgG antibody was found to be significantly higher in the serum of critical female patients compared to males^82^. Thus, it is conceivable that a combination of immune suppressive effects and the oncogenic role of androgen/AR axis could potentially augment the magnitude of SARS-CoV-2 infection in men with prostate cancer, resulting in an unfavorable cancer outcome.

Cellular mediators of COVID-19 and potential crosstalk with prostate cancer: Another factor connecting COVID-19 infection and prostate cancer is the high expression of TMPRSS2 in prostate cancer. SARS-CoV-2 entry into the host cell depends on the serine protease activity of TMPRSS2^83–87^. Cells overexpressing TMPRSS2 are susceptible to SARS-CoV-2 infection^85^. Compared to low levels of ACE2 expression as analyzed in multiple tissues, TMPRSS2 shows broad tissue distribution with higher expression (see refs. ^87,88^ and Supplementary Fig. 1). TMPRSS2 is highly expressed in urogenital organs like prostate, seminal vesicles, testis, epididymis, and kidney^89^. TMPRSS2 expression is regulated by androgen/AR signaling^90–95^. In the prostate, it is expressed primarily in the luminal cells and is fused with ETS transcription factors, predominantly ERG and ETV1, in a large proportion of prostate cancers^96–99^. Gene fusions between TMPRSS2 gene and ERG are evidenced in approximately 50% of prostate cancers^96–99^. TMPRSS2 is expressed at high levels in both primary, as well as in, metastatic prostate cancers. Several studies have demonstrated the presence of AR-responsive elements (AREs)/ARBSs in the promoter enhancer, as well as in the intronic region of the TMPRSS2 gene^90–95^. A significantly positive correlation for the expression of AR and TMPRSS2 was seen in both primary and metastatic castrate resistant prostate cancer^100^. These findings indicate that more androgens could signify greater TMPRSS2 expression, which could potentially increase vulnerability to SARS-CoV-2. Interestingly, a recent study shows that ACE2 expression is higher in males and may be regulated by androgen/AR signaling. The study found evidence of AR and ACE2 co-expression in multiple tissues^57^. Further, single cell analysis showed the presence of ACE2 expressing cell clusters within the prostate and testis^57^. It appears that together both TMPRSS2 and ACE2 might be under hormonal control. More validation studies are needed to confirm the direct role of androgens in the regulation of ACE2.

COVID-19 related inflammation, TMPRSS2, and prostate cancer: Recurrent gene arrangements are common in prostate cancer, and catastrophic chromosome rearrangements are a hallmark of prostate carcinogenesis^101–104^. Recent studies have shown that inflammation-induced oxidative stress is an essential driver of oncogenic TMPRSS2-ERG fusions^105^. Treatment of prostate cancer cells with TNFα produced a robust inflammatory response that resulted in DNA breaks and de novo genomic arrangements mediated by a non-homologous end joining (NHEJ) process^105^.

Work from others and ongoing studies in our laboratory suggest that systemic and pelvic inflammation are critical factors for prostate cancer progression and that proinflammatory cytokines (e.g., IL-1, IL-6, IL-8, and MCP-1) can accelerate the progression of existing disease (manuscript in preparation)^106–108^. Interestingly, elevated levels of IL-1 and IL-6 are also associated with SARS-CoV-2 infection^109,110^. The levels of TNF alpha cytokine are elevated in the blood and tissue of patients with SARS-CoV-2 infection and are a major contributor to immune pathology in conjunction with IL-1β, IL-6, IL-8, and MCP-1^49,111^.

Due to a higher incidence of COVID-19 in males, the direct role of TMPRSS2 gene in the infection, and the potential association between inflammation and prostate cancer, studies that critically address the impact of inflammatory cytokines TNF, IL-1β, IL-6, and IL-8 on prostate cancer can help us to better understand the impact of COVID19 on prostate cancer etiology and carcinogenesis.

Emerging evidence suggests that SARS-CoV-2 infections and complications can adversely affect the urinary tract and genitourinary organs^89^ such as the kidney and testes. Recent studies suggest that SARS-CoV-2 in the testes may result in a delayed clearance and persistent viral infections in males^56^. SARS-CoV-2 has been detected in semen in addition to saliva, urine, stool, blood, and the gastrointestinal tract^112,113^.

COVID-19 related inflammation from adjacent tissues may augment prostate cancer: Inflammation is the driver of prostate cancer carcinogenesis^114^. Anatomically, the prostate is positioned below the bladder and lies close to the seminal vesicles and the rectum. For these reasons, the prostate may be vulnerable to adverse effects of the secreted inflammatory cytokine milieu that is generated as a result of SARS-CoV-2 in semen, the testes, and feces. Semen is produced by secretions from the seminal vesicle, prostate, and testes. Vas deferens, the duct that carries sperm from the testes, enters the ejaculatory duct and then passes through the prostate to the urethra. This trajectory could result in the dissemination of viral cytokines to the prostate with deleterious outcomes.

**Fig. 3:**
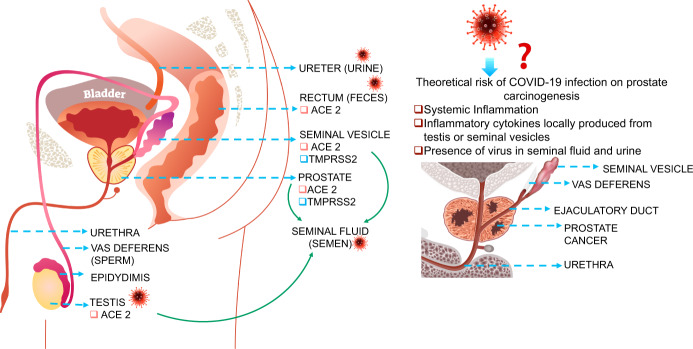
Graphical representation of the theoretical risks and potential routes of dissemination of SARS-CoV-2 to the prostate. This is based on tissue level expression of **a** TMPRSS2 and ACE2; and **b** the presence of virus in body fluids such as urine, semen, and feces. Systemic or tissue derived inflammation, during COVID-19, has the potential to accelerate pre-existing prostate cancer resulting in an aggressive phenotype and therefore represents a potential risk factor for prostate cancer patients.

It is interesting to note that seminal vesicles express ACE2 and TMPRSS2^89,115,116^ and could be a direct target of SARS-CoV-2 infection that could result in an inflammatory mileu in the vicinity of the prostate. Conceptually this can occur through systemic circulation via the arteries that feed the prostate and seminal vesicles, or via ducts that connect the prostate and seminal vesicles, and could have deleterious effect on prostate cancer progression (Fig. 3). Further, based on a recent study^57^ that showed ACE2 and TMPRSS2 are expressed in prostate cells, it is conceivable that prostate may be a direct target of SARS-CoV-2 associated inflammation and adverse pathogenesis of COVID-19.

**Fig. 4:**
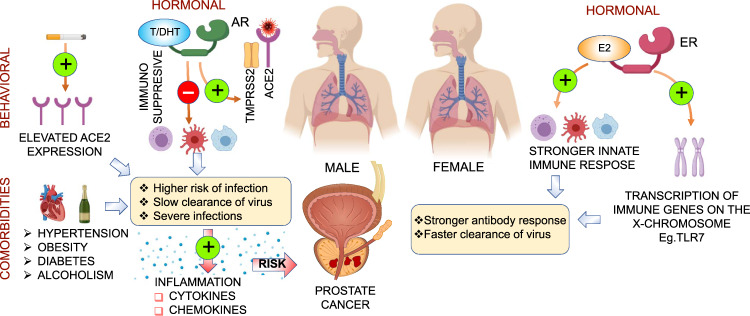
Sex differences in COVID-19. Illustration highlights potential biological (hormone signaling, immunological) and behavioral differences between males and females that contribute to sex divergence in response to SARS-CoV-2. Estrogens have protective functions that contribute to stronger innate immune response in females resulting in faster clearance of virus loads. Androgens and AR regulate tissue level expression of TMPRSS2; serine protease involved in SARS-CoV-2 infection combined with immunosuppressive effects and comorbidities can contribute to the severity of disease in males. Inflammation, an outcome of COVID-19 associated with severe infection, is a potential risk factor for prostate cancer and can augment progression of active disease.

While these hypotheses are currently less well understood, they suggest potential risks and routes of dissemination of infection in the context of prostate cancer. They will require in-depth molecular validation and testing. The potential biological (hormone signaling, immunological) and behavioral differences between males and females that contribute to sex divergence in response to SARS-CoV-2 and the inferred link between COVID-19 and prostate cancer have been summarized in Fig. 4.

### Current therapeutic options for COVID-19 patients and COVID-19 patients with prostate cancer

Drug repurposing: urgent clinical developments for the treatment of COVID-19: In the context of the current global pandemic, there is an unmet need for novel therapeutics to treat COVID-19. The focus has recently shifted toward the repurposing of existing drugs, including antivirals. To date, 1460 clinical trials are currently active worldwide, including 667 on therapeutic interventions, 199 of which are underway in the United States. More than 20 drugs are currently under investigation, most notably Remdesivir (Gilead; RNA-dependent RNA-polymerase inhibitor and broad spectrum antiviral), hydroxychloroquine (antimalarial), Lopinavir/Ritonavir (HIV-1 virus protease inhibitors), Tocilizumab (IL-6 inhibitor)^117^, Sarilumab (IL-6 antagonist), Favilavir (antiviral), inhaled nitric oxide, corticosteroids, and many more^118,119^. Remdesivir showed reduction in COVID-19 symptoms compared to placebo in an 800-patient, phase-II trial, launching the drug into phase III trials by Gilead and moving the drug one step closer toward FDA approval^120,121^. Remdesivir was available through compassionate use authorization by the FDA. A multicenter study showed clinical improvement in 68% of patients who received Remdesivir under compassionate use^122^. Rapamycin and Metformin are geroprotective drugs that have also been repurposed for prevention of SARS-CoV-2 infections in elderly people^123^. While Rapamycin, an mTOR inhibitor has been an effective antiviral agent in MERS-CoV infection^124^ as well as in patients with H1N1 pneumonia^125^, Metformin, is widely used as an oral diabetic medicine.

In addition to the drugs cited above, several other potential candidates are in various stages of development. Another RNA-dependent RNA-polymerase inhibitor, Favipiravir, shows promising results^126,127^. One surprising candidate is Famotidine, an H2 receptor antagonist commonly prescribed for heartburn^128^. The triple antiviral therapy lopinavir-ritonavir-interferon beta1b was shown to significantly reduce the duration of viral shedding by 5 days in COVID-19 positive patients^129^.

Efforts targeted at developing a vaccine are also underway, including mRNA-1273 and mRNA-1647 (Moderna, United States), INO-4800 (Inovio), Ad5-nCoV (CanSino Biologics), ChAdOx1 nCoV-19 (Vaccitech, United Kingdom), LV-SMENP-DC (Shenzhen Geno-Immune Medical Institute, China), CD24Fc (Institute of Human Virology, United States), and nCoV-specific aAPC (Shenzhen Geno-immune Medical Institute, China), with more than one in phase I development^130^. Most of these vaccines are protein-based, and there appears to be a trend to produce more varieties of vector-based vaccines (Ad5-nCoV, LV-SMENP-DC, nCoV-specific aAPC). Very few, such as the vaccine by Moderna, are nucleic acid-based (mRNA-1273, INO-4800)^131^. Long-term outcomes are unclear, particularly in younger age groups and immune compromised individuals. As was the case with SARS-CoV, exposure to attenuated forms of SARS-CoV-2 may lead to hypersensitivity-induced exacerbation of COVID-19 symptoms, or stimulate viral mutation, propagating further outbreaks^132^. Additionally, timing for optimal effect, commercial availability, and production scalability of the vaccine are major issues during a pandemic^133^. Angiotensin receptor 1 (AT1) blockers (Losartan, Telmisartan) have been proposed as an alternative preventive therapy to vaccination^134^ and are currently in phase I testing (NCT04335123).

Prostate Cancer therapies repurposed for COVID-19 patients: Antiandrogens and TMPRSS2. Because TMPRSS2 is androgen regulated, several studies have advocated for the use of antiandrogens and androgen deprivation therapies (ADT) as a potential therapeutic option in COVID-19, suggesting possible protection against SARS-CoV-2 infection^46,79,135,136^. Consistent with this idea another study proposes that the severity of COVID19 may be influenced by androgen sensitivity^79,137^. Men with severe COVID-19 showed androgenetic alopecia, a clinical condition due to over expression of androgens and AR signaling^137^. A recent study in Italian patients in COVID-19 wards found that prostate cancer patients on androgen deprivation therapy were four times less likely to get infected than prostate cancer patients not taking androgen deprivation therapy, and five times less likely to contract the disease compared to patients with other cancer types^46^. A trial on the use of Bicalutamide (antiandrogen) on COVID-19 is underway in Baltimore, Maryland. (NCT04374279). With the hope of abrogating androgen dependent TMPRSS2 activation in COVID-19 male patients, the U.S. department of Veterans Affairs (VA) launched a phase II trial for the use of the hormone suppresser, Degarelix (GnRH analog, blocks luteinizing hormone and thereby reduces androgens) for COVID-19 male patients^138^. Despite the potential benefit of antiandrogens for the treatment COVID-19, a recent study cautions against the use of antiandrogens in COVID-19 patients and indicates that the treatment may be ineffective against lung TMPRSS2^139^. In a related study using mouse models, antiandrogens were shown to be ineffective in decreasing lung TMPRSS2 expression and high AR expression in the lungs suggested TMPRSS2-independent androgen receptor mediated regulation in males^140^. Interestingly, in lung A549 cells TMPRSS2 is also regulated by the glucocorticoid, Dexamethasone^141^. Glucocorticoid receptor (GR) inhibitors have been studied in the context of prostate cancer and have been shown to increase the therapeutic benefit of antiandrogens. For these reasons, repositioning GR inhibitors for the treatment of COVID-19 patients may be a viable option.

An alternative to antiandrogens, TMPRSS2 function can be altered by blocking its enzymatic activity^136^. Examples of drugs in this category include TMPRSS2 protease inhibitors such as Bromhexine (mucolytic agent), Camostat and Nafamostat (serine protease inhibitors), and aerosolized aprotinin (antiviral). Bromhexine, Camostat, and Nafamostat are currently under investigation in more than nine clinical trials (NCT04338906, NCT04355052, NCT04353284, NCT04321096, NCT04374019, NCT04352400, NCT04355026, NCT04273763, and NCT04340349).

**Fig. 5:**
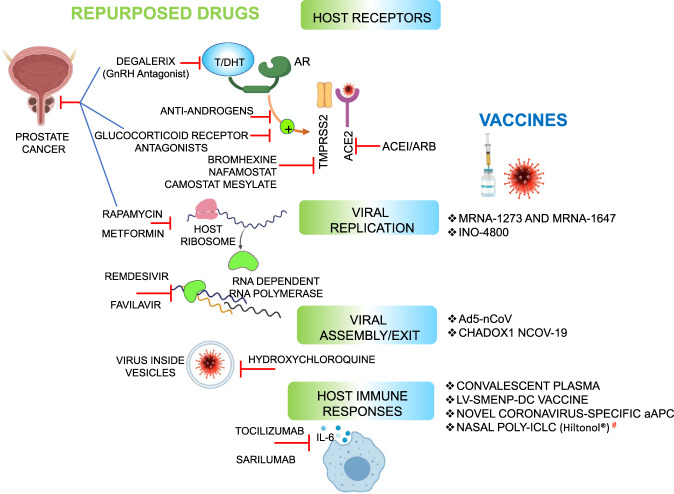
Major drugs and vaccines for COVID-19. Illustration summarizes potential drugs and vaccines for COVID-19 that target either host receptors, viral replication, virus assembly, or host immune response. Drugs that have been implicated in prostate cancer management (Degalerix, Anti-androgens, Glucocorticoid receptor antagonists, metformin, and rapamycin) appear in the blue lines. # Nasal Poly-ICLC (Hiltonol®), Oncovir Inc, is being investigated as a new therapeutic agent at Mount Sinai.

Further, based on available evidence we hypothesize that prostate cancer patients on antiandrogens may be at a lower risk of COVID-19 infection and severity. Potential drugs and novel vaccines to combat SARS-CoV-2 infection, as well as repurposed drugs that could have an impact on prostate cancer management are listed in Fig. 5.

Clinical management of COVID-19 positive prostate cancer patients: COVID-19 poses significant challenges for clinical decision-making and the management of prostate cancer patients. Physicians caring for prostate cancer patients will be expected to mitigate the risks associated with COVID-19 infections while also providing the best clinical care for patients who are dealing with decisions about biopsy, active surveillance, surgery, radiation, hormonal therapy, and chemotherapy. All of these scenarios will take place as new knowledge about, and treatments for, COVID-19 and COVID-19 as it overlaps with prostate cancer continue to evolve. In this context, a screening strategy for COVID-19 will be critical, as well as the development and implementation of a triage protocol and tools to quickly identify patients in need of immediate treatment. COVID-19 specific treatment guidelines are being developed for prostate cancer patients; nonetheless many decisions will be left to individual clinicians, especially given the uncertainty that surrounds potential new surges and the likely changes in the delivery of healthcare in the post-pandemic era.

Currently, no evidence exists on the outcomes of standard prostate cancer therapy in the setting of COVID-19, and treatment guidelines in cancer patients infected with SARS-CoV-2 are largely left up to the discretion of specialists^142^. Urologists may need to weigh the imminent risk of death from COVID-19 against the risk of death from cancer long-term.

### Future directions

Moving forward, it is essential to recognize the nuanced challenges associated with COVID-19 and prostate cancer and to adopt novel strategies that will eventually lead to better management of COVID-19 in prostate cancer patients. The true susceptibility of prostate cancer patients to COVID-19 remains unclear at this point, despite early evidence of overlapping biology and common comorbidities and requires in-depth investigation. Such timely initiatives will guide prospective research and preparedness in the post-pandemic era and if a surge of SARS-CoV-2 infections reoccurs. The particular vulnerability of older men to COVID-19 compels us to advocate for routine screening of SARS-CoV-2 infections in prostate cancer patients. Clinical trials testing the value of diverse therapeutics in COVID-19 will be ongoing, and as we learn about the mechanisms of action in COVID-19 that cause lethal disease, we can expect within the next few months to have answers to pressing questions about treatment options.

Various urology, immunology, and oncology research groups, including our own, are exploring drug repurposing and novel immunomodulation strategies to combat COVID-19. While several studies are testing BCG^143^ and convalescent plasma^144^ for prevention of COVID-19, our group is examining potential new approaches for COVID-19, including the use of polyinosinic–polycytidylic acid stabilized with polylysine and carboxymethylcellulose (Poly-ICLC (Hiltonol®) a synthetic, stabilized double-stranded RNA (dsRNA) therapeutic viral mimic and a modulator of innate and adaptive immune responses^145^. Poly-ICLC has been shown to generate a broad spectrum of innate antiviral protections^146–150^ and earlier studies have demonstrated immediate protection in a mouse model of SARS-CoV with lethal infection^151^. Due to the severity of the pandemic in males, a clinical trial in men at risk or diagnosed with prostate cancer and interacting with health care workers for management of their prostate cancer is timely and of critical significance. We are currently exploring the feasibility of a clinical trial of nasal Poly-ICLC (Hiltonol®) for COVID-19 in men (age group ≥40) who are at high risk of contracting COVID-19 and with factors/comorbidities associated with higher mortality. An ongoing dose-escalation study (NCT03262103) to determine a safe dose and schedule of intratumoral (IT) plus intramuscular (IM) Poly-ICLC (Hiltonol®) injections prior to radical prostatectomy in patients with prostate cancer has shown that it was well tolerated in patients at high risk of recurrence (*n* = 6; Cohorts 1&2).

As the pandemic continues the ongoing work is focused on the development and performance of clinical trials in COVID-19 patients and the repurposing of existing drugs to establish their therapeutic efficacy. Precise and sophisticated molecular and biological knowledge should drive future clinical work, which can have near-term therapeutic benefit for patients diagnosed with COVID-19.

It is our obligation as researchers and clinicians to provide rigorous evidence-based knowledge during this global health crisis and to develop therapeutic options to address COVID-19 as the pandemic evolves. The work to combat the virus and prostate cancer is urgent and critical.

## Supplementary information

Supplementary Information
