# Supplementary material for: Sex differences in SARS-CoV-2 infection rates and the potential link to prostate cancer
Source: Commun Biol. 2020 Jul 8;3:374. doi: 10.1038/s42003-020-1088-9 (PMC7343823; doi:10.1038/s42003-020-1088-9)
Supplement: Supplementary file 1 — Supplementary Information [file 42003_2020_1088_MOESM1_ESM.pdf]

Supplementary Figure 1

Expression data for TMPRSS2 and ACE2 from Genotype-Tissue Expression (GTEx) project (https://gtexportal.org) is given below.

A. Median expression of TMPRSS2 in multiple tissues is shown. The expression data described here were obtained from https://gtexportal.org/home/gene/TMPRSS2 the GTEX portal on 5/21/2020 and Data Source: GTEx Analysis Release V8 (dbGaP Accession phs000424.v8.p2)
https://gtexportal.org/home/gene/TMPRSS2
Gene expression for TMPRSS2 (ENSG00000184012.11)

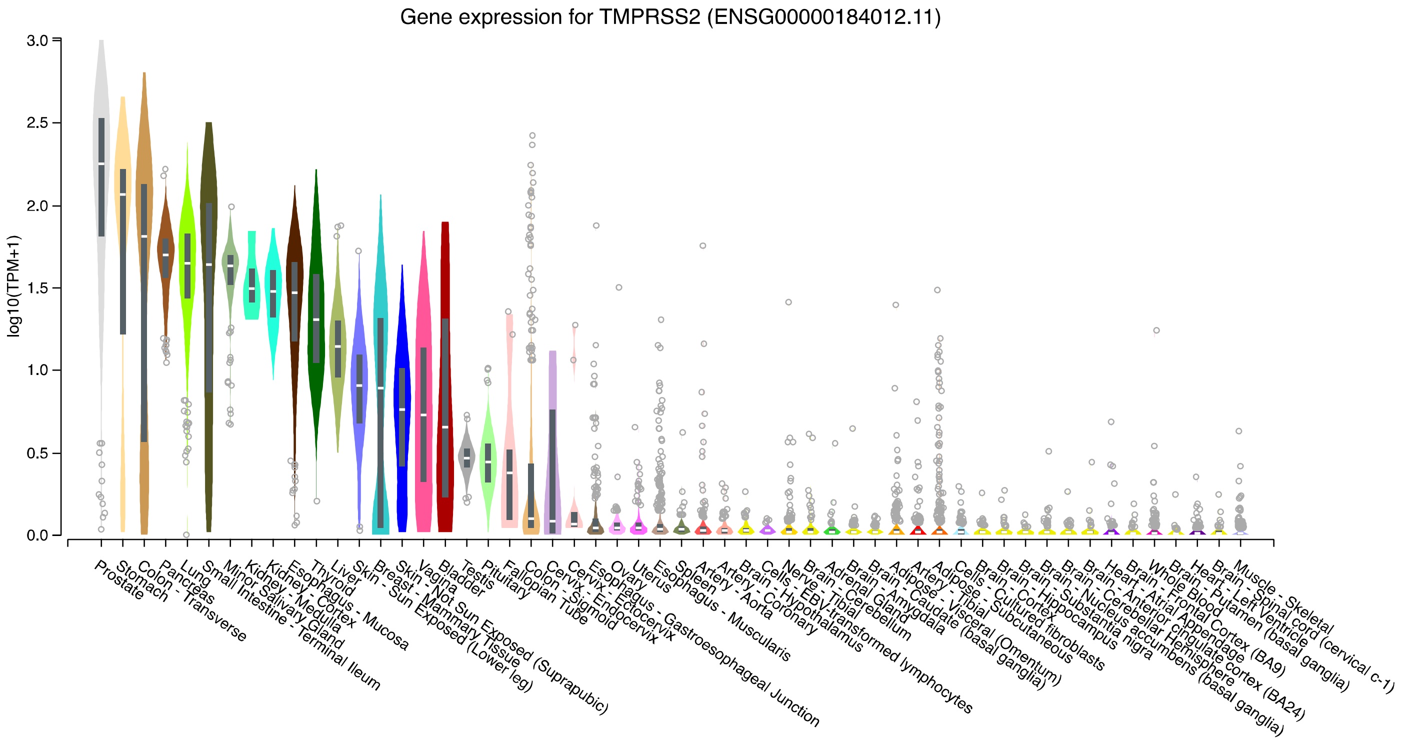

B. Median expression of TMPRSS2 in multiple tissues separated by sex is shown. The expression data were obtained from https://gtexportal.org/home/gene/TMPRSS2 the GTEX portal on 5/21/2020 and Data Source: GTEx Analysis Release V8 (dbGaP Accession phs000424.v8.p2)

https://gtexportal.org/home/gene/TMPRSS2
Gene expression for TMPRSS2 (ENSG00000184012.11)

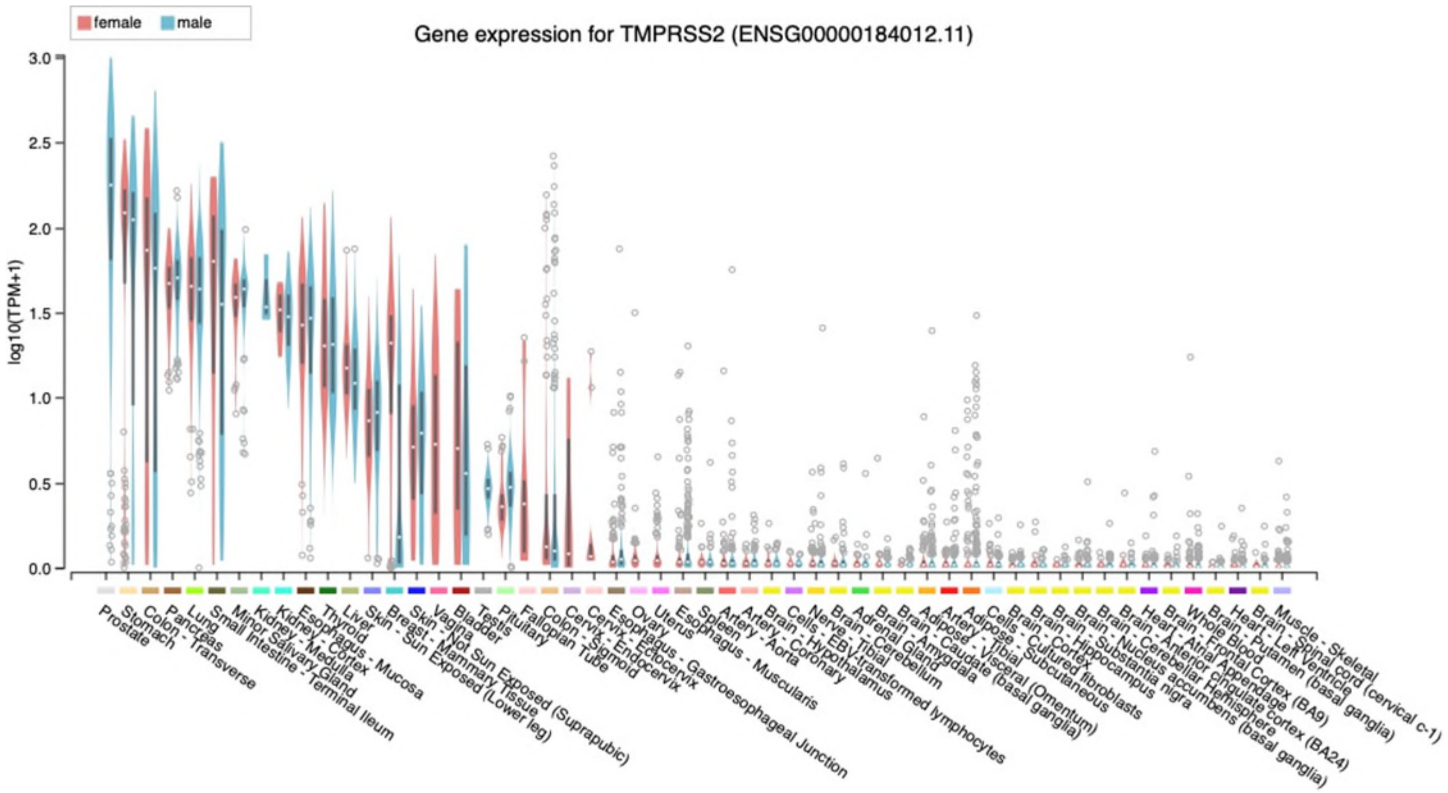

C. Median expression of ACE2 in multiple tissues is shown. The expression data described here were obtained from https://gtexportal.org/home/gene/ACE2 the GTEX portal on 5/21/2020 and Data Source: GTEx Analysis Release V8 (dbGaP Accession phs000424.v8.p2)

https://gtexportal.org/home/gene/ACE2
Gene expression for ACE2 (ENSG00000130234.10)

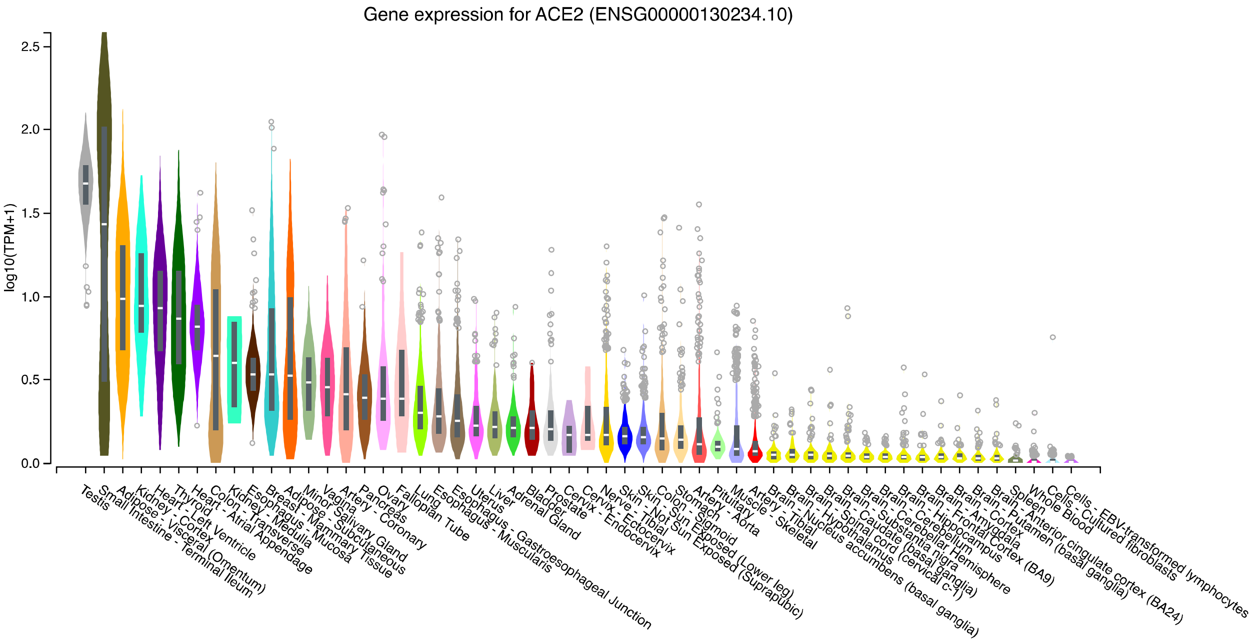

D. Median expression of ACE2 in multiple tissues separated by sex is shown. The expression data described here were obtained from https://gtexportal.org/home/gene/ACE2 the GTEX portal on 5/21/2020 and Data Source: GTEx Analysis Release V8 (dbGaP Accession phs000424.v8.p2)

https://gtexportal.org/home/gene/ACE2
Gene expression for ACE2 (ENSG00000130234.10)

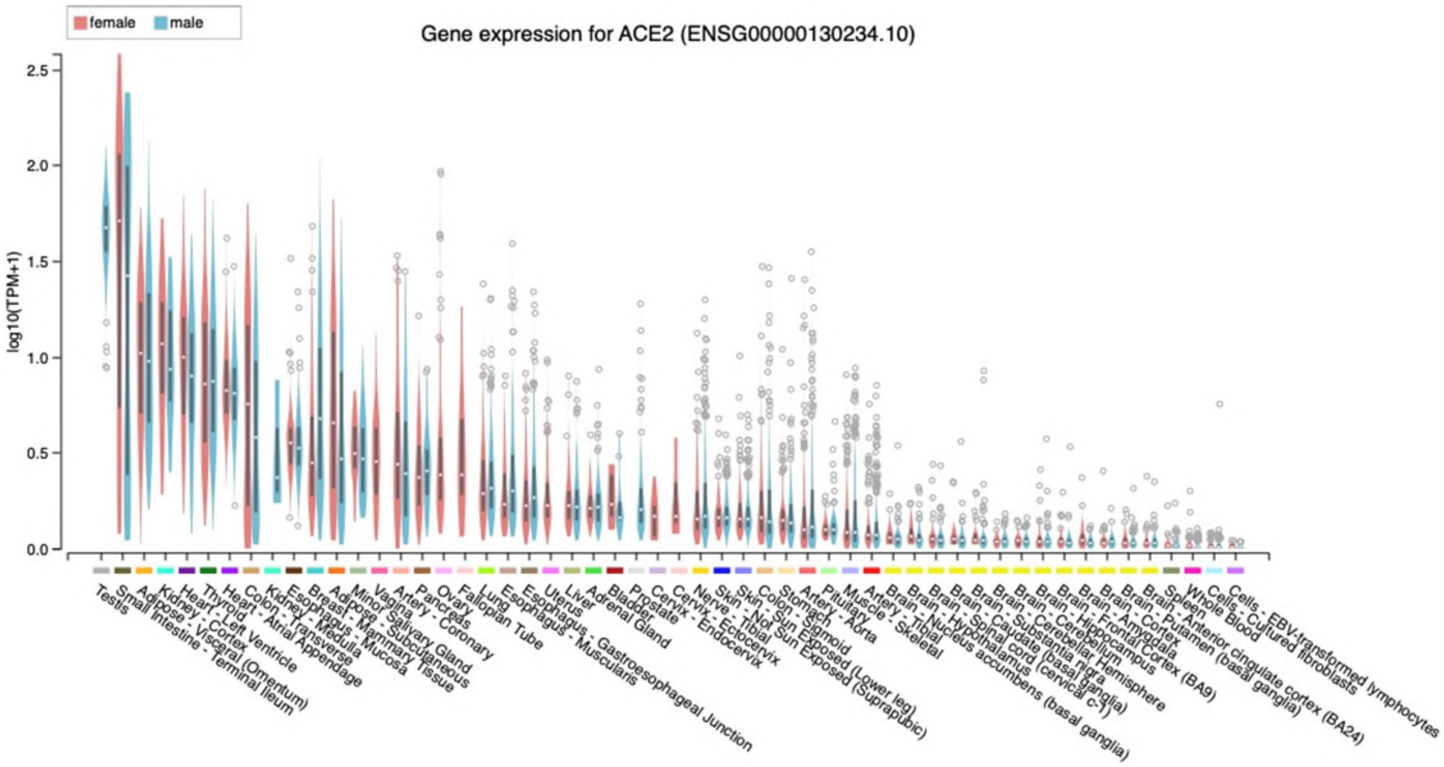

Acknowledgement: The Genotype-Tissue Expression (GTEx) Project was supported by the Common Fund of the Office of the Director of the National Institutes of Health, and by NCI, NHGRI, NHLBI, NIDA, NIMH, and NINDS.
